# Enhanced sensitivity of celecoxib in human glioblastoma cells: Induction of DNA damage leading to p53-dependent G_1 _cell cycle arrest and autophagy

**DOI:** 10.1186/1476-4598-8-66

**Published:** 2009-08-25

**Authors:** Khong Bee Kang, Congju Zhu, Sook Kwin Yong, Qiuhan Gao, Meng Cheong Wong

**Affiliations:** 1 Brain Tumour Research Laboratory, Division of Medical Sciences, Humphrey Oei Institute of Cancer Research, National Cancer Centre, 11 Hospital Drive, 169610, Singapore

## Abstract

**Background:**

Selective cyclooxygenase (COX)-2 inhibitors elicit anti-proliferative responses in various tumours, however the underlying anti-tumour mechanisms are unclear. Mutational inactivation of the tumour suppressor p53 gene is frequent in malignant gliomas. The role of p53 mutation in the anti-tumour responses of the selective COX-2 inhibitor celecoxib in human glioblastoma cells is unknown. In this study, we used human glioblastoma cells with various p53 status; U87MG (with high and low p53 functional levels), LN229 (functional p53) and U373MG (mutant p53) cells. Inhibition of p53 was achieved in U87MG cells transfected with E6 oncoprotein (U87MG-E6) and treated with pifithrin-α, a reversible inhibitor of p53 (U87MG-PFT). We investigated whether the anti-glioblastoma responses of celecoxib were p53-dependent, and whether celecoxib induced DNA damage leading to p53-dependent G_1 _cell cycle arrest, followed by autophagy or apoptosis.

**Results:**

Our findings demonstrated that celecoxib concentration-dependently reduced glioblastoma cell viability, following 24 and 72 hours of treatment. Inhibition of functional p53 in glioblastoma cells significantly reduced the anti-proliferative effect of celecoxib. In U87MG cells, celecoxib (8 and 30 μM) significantly induced DNA damage and inhibited DNA synthesis, corresponding with p53 activation. Celecoxib induced G_1_-phase cell cycle arrest, accompanied with p21 activation in U87MG cells. Cell cycle progression of U87MG-E6 and U87MG-PFT cells was not affected by celecoxib. In parallel, celecoxib induced G_1 _cell cycle arrest in LN229 cells, but not in U373MG cells. Autophagy was induced by celecoxib in U87MG and LN229 cells, as shown by the significantly greater population of acridine orange-stained cells and increased levels of LC3-II protein (in comparison with non-treated controls). Celecoxib did not induce significant autophagy in U87MG-PFT, U87MG-E6 and U373MG cells, which lack functional p53. Regardless of p53 status, celecoxib caused no significant difference in apoptosis level of U87MG, U87MG-PFT, U87MG-E6 and U373MG cells.

**Conclusion:**

Our findings reveal that p53 increases human glioblastoma sensitivity to celecoxib. Celecoxib inhibits glioblastoma cell viability by induction of DNA damage, leading to p53-dependent G_1 _cell cycle arrest and p53-dependent autophagy, but not apoptosis.

## Introduction

Despite conventional therapy of surgical resection, radiotherapy and chemotherapy, the median survival of malignant glioma patients remain poor. Most patients with glioblastoma multiforme survive less than 2 years after diagnosis [1]. Therapeutic improvements are needed to extend the survival of malignant glioma patients. Cyclooxygenase (COX)-2, an isoform of COX which is the rate-limiting enzyme in conversion of arachidonic acid into prostaglandins, is inducible in the presence of cytokines and growth factors during inflammation [2]. The importance of COX-2 in carcinogenesis and brain tumour progression is highlighted by the detection of COX-2 in brain tumours [3,4] and COX-2 overexpression in gliomas associated with poor prognosis [5]. Targeting COX-2 with selective COX-2 inhibitors (NS-398, SC-236 and celecoxib) has proven effective to reduce human glioblastoma cell viability *in vitro *[4,6-9] and in rodent models [6,9-11]. Celecoxib is the only selective COX-2 inhibitor approved by the FDA for adjuvant treatment of patients with familial adenomatous polyposis.

The molecular events underlying the anti-tumour properties of COX-2 inhibitors are not fully understood. Several mechanisms have been proposed in various tumour models. COX-2 inhibition by celecoxib induces G_1 _cell cycle arrest, corresponding with activation of G_1_-phase cyclin-CDK inhibitors, p21 and p27 [12-14]. Celecoxib activates apoptotic proteins BAD, caspases and PARP, followed by cell apoptosis and reduced tumour cell proliferation [9,13-17]. Anti-tumour mechanisms of COX-2 inhibitors also include inhibition of tumour angiogenesis [18], inhibition of prostaglandin-induced immunosuppressive activity [19] and increased DNA damage/reduced DNA repair capacity [20]. Peroxidation of arachidonic acid into prostaglandins by COX generates reactive oxygen species and free radicals, which induce DNA damage and tumourigenicity [21]. Inhibition of COX by COX inhibitors aspirin [22], nimesulide [23], rofecoxib and celecoxib [24] protects DNA from oxidative damage by scavenging hydroxyl radicals and superoxide *in vitro *in non-tumour models. However, prevention of DNA damage by COX inhibitors has not been reported in tumour cells. In contrast, aspirin significantly induces DNA damage of HT-29 human colon carcinoma [25], whereas celecoxib causes DNA damage in MCa-35 murine mammary and A549 human lung cancer cells [26]. Whether COX-2 inhibitors induce DNA damage in glioblastoma cells is unclear.

Mutational inactivation of the tumour suppressor gene p53 (a regulator of cell growth and death) is frequently found in human tumours, with p53 mutation/inactivation reported in 63–65% of high-grade gliomas [27]. Induction of DNA damage initiates a cascade of signalling with p53 activation (phosphorylation at Ser 15 and Ser 20) and subsequent transcriptional activation of p53 response genes (including p21, GADD45, BAX, PUMA, Bcl2 and NOXA), thus provoking cell cycle arrest and/or apoptosis [28]. Genotoxic stress caused by DNA-damaging agents also induce p53-dependent autophagy [29,30], the type II programmed cell death characterised by the formation of cytosolic double-membrane vesicles (autophagosomes) that engulf cellular content by digestion, when fused with lysosomes [31]. The mechanisms of p53-dependent induction of autophagy are not fully understood, but are thought to involve both the transcription-independent functions (e.g. activation of the nutrient energy sensor AMP kinase) and transcription-dependent functions (e.g. upregulation of mTOR inhibitors PTEN and TSC1, or p53-regulated autophagy and cell death gene DRAM) [30,32]. Anti-tumour mechanisms by COX inhibition have been shown to be either p53-dependent [33,34] or p53-independent in various cancer and non-cancer cells. The anti-proliferative mechanism of COX-2 inhibitors underpin by autophagy induction in tumours is unclear. To date, only one recent report suggests that celecoxib induces both autophagy and apoptosis, mediated by P-glycoprotein independent of p53 mechanisms, in hepatocellular carcinoma cells [35]. The role of p53 in celecoxib-induced autophagy and celecoxib-induced anti-proliferative responses clearly needs to be verified.

In this study, we investigated (a) whether the anti-proliferative response induced by celecoxib was dependent on the presence of functional p53 and b) whether celecoxib-induced DNA damage resulted in p53-dependent G_1 _cell cycle arrest, followed by apoptosis or autophagy. We studied the effect of celecoxib in human glioblastoma cells with various p53 status; U87MG cells with high and low levels of p53 [by both genetic (oncoprotein E6) and pharmaceutical intervention (pifithrin-α)], LN229 (with wild type p53 function despite a p53 mutation in the coding sequence [36]) and U373MG (with mutant p53) cells. Our findings show that the anti-proliferative sensitivity of celecoxib is dependent on p53 in human glioblastoma cells. We further demonstrate that celecoxib enhances glioma cytotoxicity by induction of DNA damage and p53-dependent G_1 _cell cycle arrest, followed by p53-dependent autophagy but not apoptosis.

## Results

### Celecoxib concentration-dependently inhibited human glioblastoma cell viability, with enhanced anti-proliferative response by the presence of functional p53

**Figure 1:**
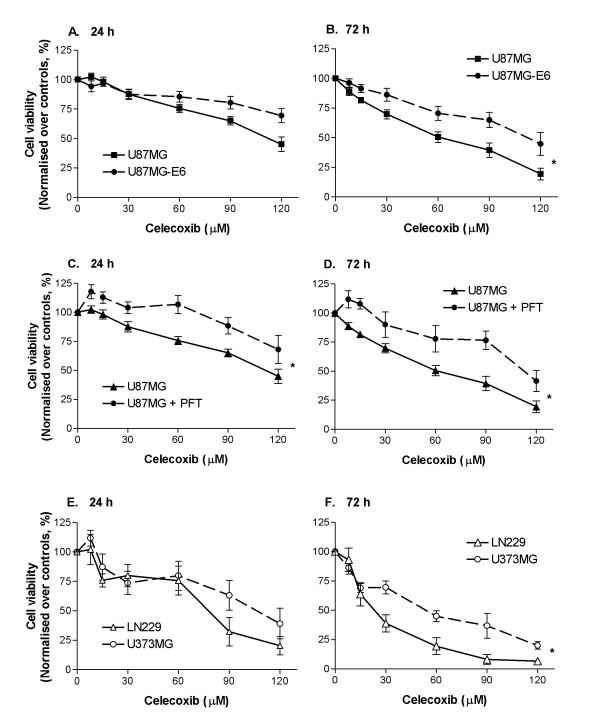
**Concentration-dependent response of celecoxib on viability of human glioblastoma cells**. Cells were treated with a series of celecoxib concentrations for 24 (A, C, E) and 72 (B, D, F) hours. In some cases, U87MG cells were pre-treated with PFT (25 μM) for 30 minutes prior to celecoxib treatment. Cell viability was measured by MTT assays and presented as absorbance readings at 570 nm normalised against controls. Values are expressed as mean ± S.E.M (*n *= 3–10). *P < 0.05 significantly different between two concentration-dependent response curves (2-way ANOVA).

Celecoxib concentration-dependently reduced the viability of human glioblastoma cells U87MG, which contains wild-type p53 (Figure 1A–D). To determine whether the anti-proliferative response to celecoxib was dependent on p53, we first compared the effect of celecoxib on viability of U87MG-E6 and U87MG cells. Viral oncoprotein E6 inhibits p53 function by abrogating specific DNA binding and transactivation of p53, sequestering p53 into the cytoplasm and accelerating its degradation [37]. Inhibition of p53 by oncoprotein E6 reduced the sensitivity of U87MG cells to celecoxib, as shown by the enhanced U87MG-E6 cell viability following celecoxib treatment, compared with non-transfected U87MG cells (Figure 1A–B). Following 72 hours of celecoxib treatment, U87MG-E6 cells were significantly more viable than U87MG cells (*P *< 0.05, Figure 1B). The prerequisite of p53 to protect U87MG cells from the anti-proliferative effect of celecoxib was confirmed with U87MG cells treated with PFT (U87MG-PFT). PFT-α inhibits p53 by reversibly blocking p53-transcriptional activation [38]. Inhibition of p53 by PFT significantly reduced sensitivity of U87MG cells to celecoxib, with enhanced U87MG-PFT cell viability at 24 and 72 hours following celecoxib treatment, compared with untreated U87MG cells (*P *< 0.05, Figure 1C–D).

The p53-dependent anti-proliferative response induced by celecoxib was also shown in LN229 and U373MG glioblastoma cells. Celecoxib (24 and 72 hours treatment) inhibited viability of LN229 and U373MG cells in a concentration-dependent manner (Figure 1E–F). At 72 hours of celecoxib treatment, U373MG cells (with mutant p53) were significantly more viable than LN229 cells (with functional p53; *P *< 0.05, Figure 1F). These results parallel the enhanced anti-proliferative responses of celecoxib in U87MG cells (high level of functional p53), compared with U87MG-E6 and U87MG-PFT (low functional level of p53), thus verifying a p53-dependent anti-proliferative response induced by celecoxib. In subsequent experiments, we tested the effect of celecoxib at 8 μM, a concentration equivalent to human plasma concentration following consumption of 800 mg/kg celecoxib daily (the FDA approved dosage for familial adenomatous polyposis [39]), as well as at 30 μM, a lower than EC_50 _concentration.

### Celecoxib activated p53

**Figure 2:**
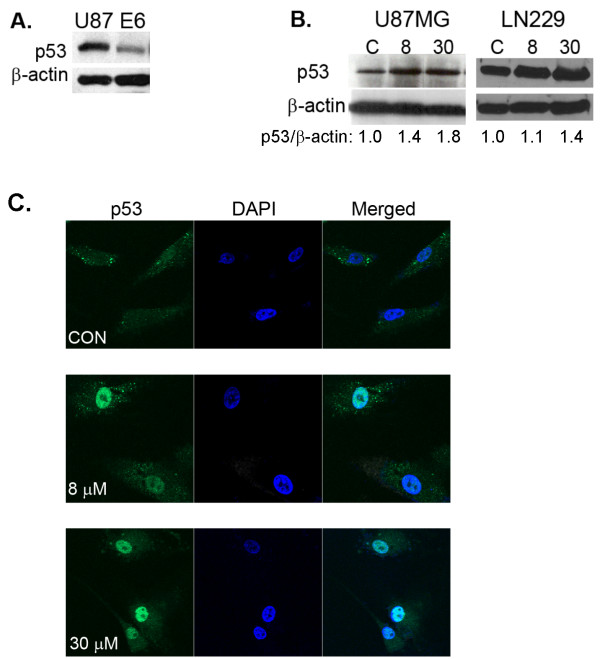
**Effect of celecoxib on p53 protein expression and localisation**. U87MG and U87MG-E6 cells were analysed for basal p53 expression (A). U87MG and LN229 cells were treated with celecoxib (18 hours), followed by immunoblotting for detection of p53. β-actin was used as loading controls. p53 expression is presented normalised over β-actin of same samples. C, controls; 8 and 30, treated with celecoxib 8 and 30 μM, respectively (B, *n *= 4–5). Representative images of celecoxib-treated (18 hours) U87MG cells immunostained with anti-p53 FITC and DAPI are shown (C, *n *= 3).

We verified that stable transfection of U87MG cells with oncoprotein E6 inhibited p53 protein expression (Figure 2A). In U87MG and LN229 cells (both with functional p53), we analysed whether celecoxib activated p53 with resultant p53-dependent anti-proliferative effects. Western blot analysis showed that celecoxib enhanced total p53 protein expression in a concentration-dependent manner in U87MG and LN229 cells (Figure 2B). Activation of p53 by celecoxib was verified by translocation of p53 from cytoplasm into nucleus when U87MG cells were treated with celecoxib (8 and 30 μM, 18 hours treatment) compared with untreated controls (Figure 2C).

### Celecoxib caused p53-dependent G_1_ cell cycle arrest, accompanied with p21 activation

**Figure 3:**
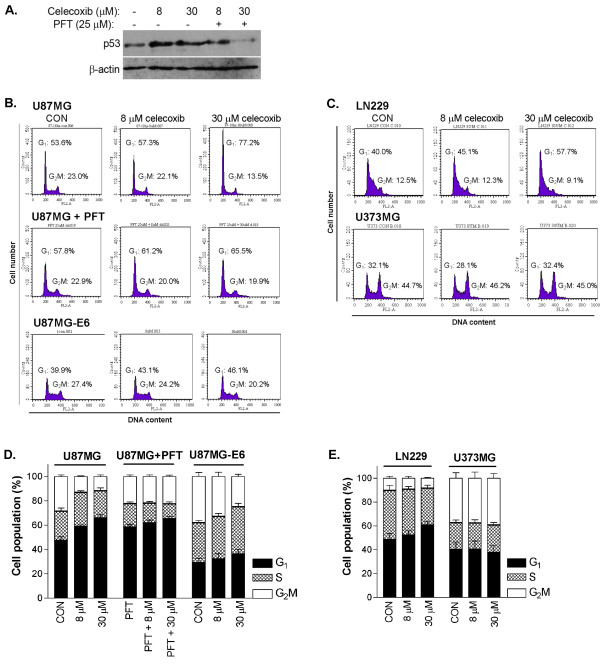
**Effect of celecoxib on p53-dependent cell cycle progression**. Cells were synchronised in serum-free medium for 48 hours and then treated with celecoxib (8 and 30 μM) in medium containing 10% FBS for 18 hours. In some cases, U87MG cells were pre-treated with PFT (25 μM) prior to celecoxib treatment. Cells were collected for protein extraction and cell cycle analysis. Following release of cells from starvation, the effect of celecoxib on p53 protein expression in U87MG and PFT-treated U87MG cells is shown (A). For cell cycle analysis, cells were fixed, stained with propidium iodide and analysed by flow cytometry. Representative histograms of cell cycle progression (B-C) and quantitative analysis of percentage gated cells at G_1_, S and G_2_M phases (D-E) are shown. All values are expressed as mean ± S.E.M, *n *= 5–8.

We analysed the human glioblastoma cells to determine whether activation of p53 by celecoxib led to cell cycle arrest. We synchronised glioblastoma cells in serum-free media for 48 hours, with resultant 75.7 ± 1.6% of U87MG cells and 82.3 ± 1.7% of U87MG-E6 cells, being arrested at G_0 _phase. Thereafter, starved cells were released from serum-free condition and treated with celecoxib for 18 hours in medium containing 10% FBS. Following release from starvation, celecoxib activated p53, as shown by the enhanced total p53 expression in U87MG cells. Addition of PFT inhibited celecoxib-induced p53 expression (Figure 3A). At 18 hours following release from starvation, cell cycle analysis showed that 47.8 ± 2.7% of untreated U87MG cells remained in G_1 _phase. Celecoxib (in a concentration-dependent manner) prevented U87MG cells from entering S-phase, resulting in a significantly greater population of cells at G_1_-phase, compared to untreated controls (*P *< 0.05, Figure 3B, 3D). There was reciprocal reduction of celecoxib-treated U87MG cells in S- and G_2_M-phases, compared to untreated controls.

To establish whether the celecoxib-induced G_1 _cell cycle arrest in U87MG cell was dependent on p53, we analysed the effect of celecoxib (8 and 30 μM) on cell cycle progression of U87MG-PFT and U87MG-E6 cells. PFT by itself, prevented U87MG cells from entering S-phase, as demonstrated by the greater population of cells at G_1_-phase (58.7 ± 2.3%) compared to the population of untreated U87MG cells at G_1 _phase (47.8 ± 2.7%; Figure 3D). PFT (25 μM), being a transient and reversible inhibitor of p53, is less efficient in blocking elevated amount of p53 (such as when p53 expression was increased during serum starvation, data not shown), resulting in a greater population of U87MG-PFT cells at G_1_phase compared to the population of U87MG cells at G_1 _phase. In parallel, Xu et al. (2005) demonstrated that PFT (20 μM) had no effect on cell cycle progression of U87MG cells [40]. Addition of celecoxib to PFT-treated U87MG cells did not affect the cell cycle progression when p53 was inhibited (*P *> 0.05, Figure 3B, 3D), suggesting a p53-dependent celecoxib-induced G_1 _cell cycle arrest in U87MG cells. Continuous inactivation of p53 by E6 in U87MG-E6 cells reduced the proportion of cells at G_1 _phase (29.5 ± 3.3%), compared with the population of U87MG cells at G_1 _phase (47.8 ± 2.7%). This is in accord with the functional role of p53 in arresting cells at G_1 _phase, as was previously shown [41]. Similar to U87MG-PFT cells, celecoxib had no significant effect on U87MG-E6 cell cycle progression (*P *> 0.05, Figure 3B, 3D), thus confirming a p53-mediated G_1 _cell cycle arrest by celecoxib in U87MG glioblastoma cells.

82.4 ± 0.9% of LN229 and 51.0 ± 3.7% of U373MG cells were arrested at G_0/1 _phase, following 48 hours of starvation in serum-free media. At 18 hours following treatment (in media containing 10% FBS), celecoxib prevented LN229 cells from entering S-phase and concentration-dependently increased the percentage population of LN229 cells in G_1 _phase, compared with untreated controls (*P *< 0.05, Figure 3C, 3E). Celecoxib had no significant effect on cell cycle progression of U373MG cells (*P *> 0.05, Figure 3C, 3E). These findings parallel the effect of celecoxib that induces G_1 _cell cycle arrest in U87MG cells, but not U87MG-E6 or U87MG-PFT cells, thus verifying an induction of p53-dependent G_1 _cell cycle arrest by celecoxib in human glioblastoma cells.

**Figure 4:**
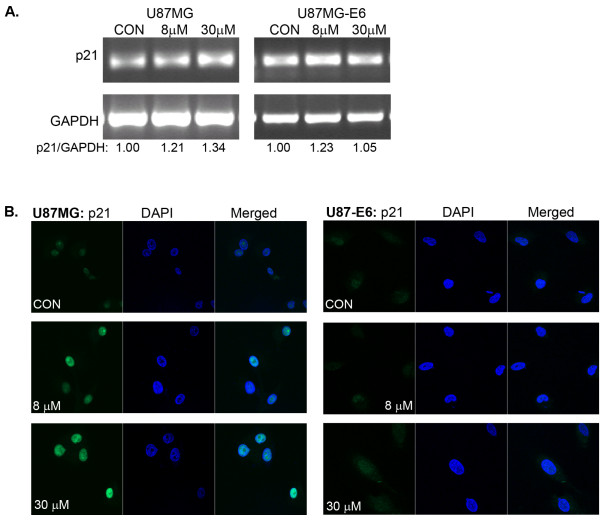
**Effect of celecoxib on p21 mRNA expression and protein localisation**. Cells were synchronised in serum-free medium for 48 hours and then treated with celecoxib (8 and 30 μM) in medium containing 10% FBS for 18 hours. Total RNA was extracted from celecoxib-treated cells, followed by RT-PCR using p21 primers. p21 mRNA expression is presented normalised over GAPDH of same samples (A, *n *= 3–5). Representative images of celecoxib-treated cells immunostained with anti-p21 FITC and DAPI are shown (B, *n *= 3).

Induction of G_1 _cell cycle arrest following DNA damage is dependent on up-regulation of CDK inhibitors such as p21, a direct transcriptional target of p53 that is strongly induced by DNA damage in cells expressing wild-type p53 [42]. We analysed whether p53-dependent G_1 _cell cycle arrest caused by celecoxib was mediated via p21 activation. Under the same synchronised cell condition where celecoxib induced p53-dependent G_1 _cell cycle arrest, our data showed that celecoxib caused a concentration-dependent increased in p21 mRNA expression in U87MG cells, but not in U87MG-E6 cells where p53 expression was depleted (Figure 4A). We verified these findings by immunocytochemistry, which demonstrated nuclear induction of p21 when U87MG cells were treated with celecoxib (8 and 30 μM; Figure 4B). In U87MG-E6 cells, celecoxib (8 and 30 μM) caused no significant changes in p21 mRNA expression (Figure 4A) and nuclear p21 protein level (Figure 4B). These data suggest that celecoxib-induced p53-dependent G_1 _cell cycle arrest is mediated by p21 activation in U87MG cells.

### Celecoxib induced p53-dependent cell autophagy but not apoptosis

**Figure 5:**
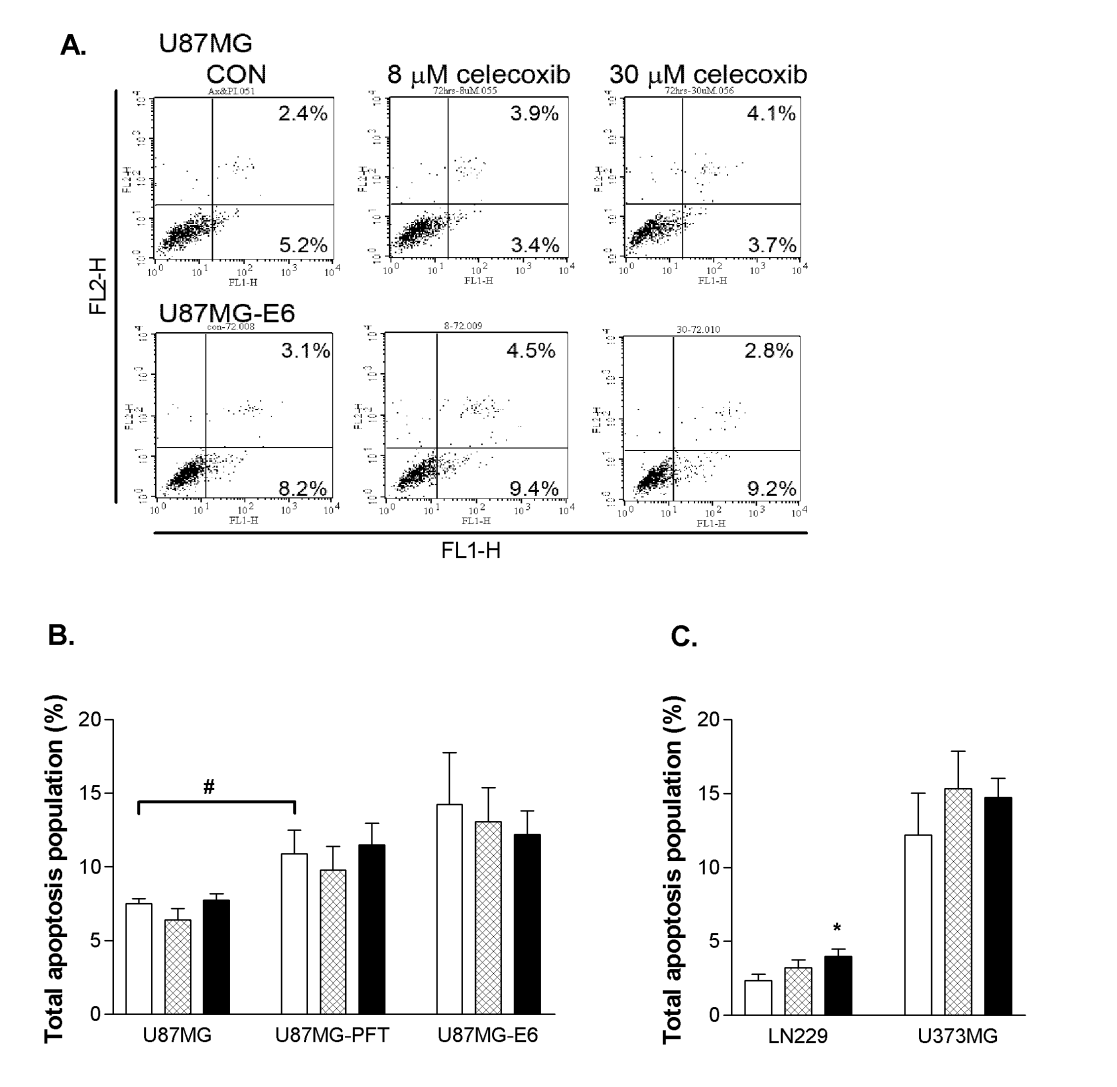
**Effect of celecoxib on cell apoptosis**. Cells were treated with celecoxib (8 and 30 μM) for 72 hours. In some cases, U87MG cells were pre-treated with PFT (25 μM) prior to celecoxib treatment. Cells were collected and stained with Annexin V-FITC and propidium iodide, followed by FACS analysis. Percentage population of early apoptosis (lower right quadrant) and late apoptosis (upper right quadrant) in celecoxib-treated U87MG and U87MG-E6 cells, stained with Annexin-FITC and propidium iodide are shown (A). Summary quantitative analyses of total apoptosis population following celecoxib treatment are presented (B-C). Values are expressed as mean ± S.E.M., *n *= 3–6. *P < 0.05, significantly different from respective controls (1-way ANOVA, Dunnett's test). ^#^*P *< 0.05, significantly different between PFT-treated and -untreated U87MG cells (Mann-Whitney U-test).

We investigated the functional consequences of celecoxib on programmed cell death type I (apoptosis) and type II (autophagy), whether celecoxib inhibited glioma proliferation by p53-dependent induction of apoptosis or autophagy. In addition to inducing apoptosis, p53 is also known to protect cells from apoptosis and necrotic cell death [43]. As such, inhibition of p53 by PFT and E6 significantly increased the apoptosis level of U87MG-PFT and U87MG-E6 cells, respectively, compared to the basal apoptosis level of U87MG cells (*P *< 0.05, Figure 5B). Similarly, the basal apoptosis level of U373MG cells (with mutant p53) was greater than LN229 and U87MG cells (both with functional p53; Figure 5B), as was also shown by others [43]. Regardless of p53 status in the glioma cells, celecoxib (8 and 30 μM, 72 hours treatment) did not cause any significant change in apoptosis population of U87MG, U87MG-PFT, U87MG-E6 and U373MG cells (*P *> 0.05, Figure 5). Celecoxib concentration-dependently increased apoptosis population of LN229 cells, from 2.4 ± 0.4% (controls) to 3.2 ± 0.5% (8 μM, *P *> 0.05) and 4.0 ± 0.5% (30 μM; *P *< 0.05, Figure 5C) of total cell population. At 72 hours treatment, celecoxib (30 μM) significantly inhibited the survival of LN229 cells to a remaining viable population of 38.9 ± 7.4% (see Figure 1F; *P *< 0.05, Student's t-test). The small 1.6% increment in apoptosis level of LN229 cells following 72 hours celecoxib treatment (30 μM) suggests apoptosis as a minor mechanism to mediate the anti-proliferative response induced by celecoxib in LN229 cells. The non-significant change in apoptosis level following celecoxib treatment in U87MG, U87MG-PFT, U87MG-E6 and U373MG cells further demonstrates that an alternative major cell death mechanism (such as autophagy) is involved in the anti-proliferative response induced by celecoxib in human glioblastoma cells.

**Figure 6:**
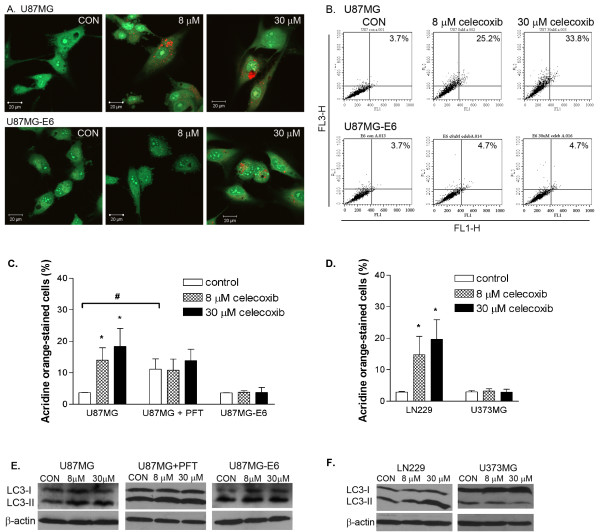
**Effect of celecoxib on p53-dependent autophagy**. Cells were treated with celecoxib (8 and 30 μM) for 72 hours. In some cases, U87MG cells were pre-treated with PFT (25 μM) prior to celecoxib treatment. Cells were collected and stained with acridine orange. Representative images of acridine orange-stained celecoxib-treated cells captured by confocal microscopy (A) and analysed by FACS (B) are shown. Summary quantitative analyses of acridine orange-stained cell populations following celecoxib treatment are presented (C-D). Values are expressed as mean ± S.E.M., *n *= 3–9. *P < 0.05, significantly different from respective controls (1-way ANOVA, Dunnett's test). ^#^*P *< 0.05, significantly different between PFT-treated and -untreated U87MG cells (Mann-Whitney U-test). Protein was extracted from celecoxib-treated (72 hours) cells and analysed for expression of the autophagy marker, LC3. Representative blots of LC3 isoforms LC3-I and LC3-II proteins in celecoxib-treated cells are shown (E-F, *n *= 3). β-actin was used as loading control.

To analyse autophagy, we used acridine orange to stain acidic vesicular organelles (AVOs) that include autophagic vacuoles [44]. In untreated U87MG cells, the cytoplasm and nucleolus fluoresced bright green and dim red. Celecoxib treatment induced the development of AVOs in U87MG cells, as shown by the concentrated fluorescence bright red acidic compartments (Figure 6A). The intensity of red fluorescence is proportional to the degree of acidity and/or volume of the cellular acidic compartment [45]. An increase in the intensity of red fluorescence was observed in U87MG cells treated with increasing concentrations of celecoxib (from 8 to 30 μM, Figure 6A–B). When the AVO staining of celecoxib-treated U87MG cells was quantified, we demonstrated that 14.0 ± 3.9% and 18.4 ± 5.7% of total cells were significantly stained with acridine orange following celecoxib treatment (8 and 30 μM, respectively), compared with untreated controls (3.8 ± 0.0%; *P *< 0.05, Figure 6C). Inhibition of p53 by PFT significantly induced autophagy of U87MG cells (*P *< 0.05, Figure 6C; [46]). Addition of celecoxib had no significant effect on the acridine orange staining of U87MG-PFT cells (*P *> 0.05, Figure 6C). In U87MG-E6 cells with reduced level of p53, development of AVOs following celecoxib treatment was not obvious (by microscopic observation, Figure 6A) and statistically non-significant (by FACS analysis; *P *> 0.05, Figure 6B, 6C). We verified the celecoxib-induced p53-dependent autophagy in U87MG cells by the changes in expression of light chain 3 (LC3)-II, an autophagosome-specific protein that is recruited to the autophagosome membrane during autophagy [47]. Celecoxib (8 and 30 μM) further induced cleavage of LC3 (conversion from LC3-I to LC3-II) in U87MG cells, in parallel with the development of AVOs following celecoxib treatment. Celecoxib (8 and 30 μM) had no effect on the level of LC3-II expression in U87MG-PFT and U87MG-E6 cells (Figure 6E).

In LN229 cells (with functional p53), celecoxib (8 and 30 μM, 72 hours treatment) significantly induced the development of AVOs (*P *< 0.05, Figure 6D), as shown by the significant increased of celecoxib-treated acridine orange-stained cells (14.8 ± 8.7% [8 μM] and 19.7 ± 7.4% [30 μM]), compared with controls (2.9 ± 1.8%). The level of autophagy induction by celecoxib in LN229 cells (4.0–5.8 fold of controls) was similar to the extent of autophagy induction in celecoxib-treated U87MG cells, which express functional p53. Celecoxib-induced autophagy response in LN229 cells was supported by the increased expression of LC3-II (Figure 6F). Celecoxib had no significant effect on the development of AVOs, or the level of LC3-II expression in U373MG cells, which contain mutant p53 (Figure 6D, 6F). These findings suggest that celecoxib induced p53-dependent autophagy rather than apoptosis in glioblastoma cells.

### Celecoxib induced DNA damage and inhibited DNA synthesis

**Figure 7:**
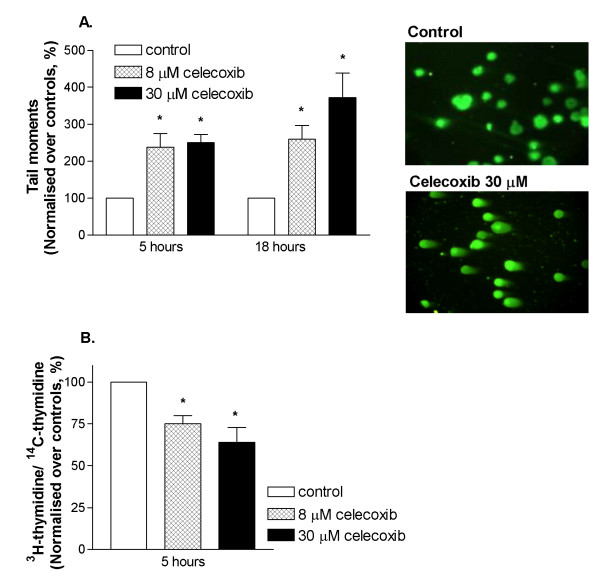
**Effect of celecoxib on DNA damage and DNA synthesis**. U87MG cells were treated with celecoxib (8 and 30 μM) and analysed for DNA damage by Comet assays, as shown by representative images and quantitated as comet tail moments (A, *n *= 5–7). DNA synthesis is presented by incorporation of [^3^H]-thymidine/[^14^C]-thymidine in celecoxib-treated cells normalised over controls (B, *n *= 3–4). *P < 0.05, significantly different from respective controls (1-way ANOVA, Dunnett's test).

To investigate the upstream events preceding p53 activation following celecoxib treatment, we analysed the effect of celecoxib on DNA damage by Comet assays under non-denaturing condition, where induction of comet tails suggests DNA double-strand breaks. Following 5 and 18 hours of treatment, celecoxib (8 and 30 μM) significantly increased comet tail moments of U87MG cells (*P *< 0.05, Figure 7A). Normalised mean tail moments by celecoxib (30 μM) at 5 and 18 hours were 259 ± 37% and 372 ± 67%, respectively, of untreated controls (Figure 7A). The effect of celecoxib on DNA synthesis was assessed by incorporation of ^3^H-thymidine into DNA during cellular S-phase. Celecoxib (8 and 30 μM, 5 hours treatment) concentration-dependently inhibited DNA synthesis of U87MG cells (by 25–36% vs. controls, *P *< 0.05, Figure 7B), corresponding with celecoxib-induced DNA damage.

## Discussion

Therapeutic targeting of glioblastoma cells with selective COX-2 inhibitors such as celecoxib has demonstrated potential [4,6-11]. However the underlying anti-proliferative mechanisms of COX-2 inhibitors remain unclear. Understanding the mechanisms underlying the anti-tumour properties of COX-2 inhibitors is needed for optimisation of therapeutic targeting by COX-2 inhibitors. In this study, we analysed the p53-dependent anti-proliferative effect induced by a selective COX-2 inhibitor, celecoxib in human glioblastoma cells. Our findings demonstrate that celecoxib induced p53-dependent G_1 _cell cycle arrest followed by autophagy, which are critical for inhibiting growth and proliferation of glioblastoma cells containing functional p53. We demonstrate insensitivity/resistance of glioblastoma cells to the anti-proliferative effect of celecoxib when p53 expression is inhibited/mutated, but enhanced cytotoxic response of celecoxib when glioblastoma cells express functional p53. Growth inhibition mediated via p53-dependent [33,34,48] and p53-independent [16,49,50] mechanisms have been reported with non-selective and selective COX-2 inhibitors in studies of tumour and non-tumour cells. In brain tumours, this finding is the first to report a p53-dependent anti-glioblastoma effect of a selective COX-2 inhibitor, which supports selective usage of celecoxib in human glioblastomas with functional p53 for enhanced anti-tumour responses.

p53 is a key molecule in DNA damage response, causing inhibition of cell proliferation by induction of cell cycle arrest, apoptosis/autophagy or senescence. The inhibitory effect of p53 on cell proliferation is due to transcriptional activation of target genes such as p21, GADD45, Bax, DR5 and PUMA [28]. In this study, inhibition of COX-2 by celecoxib [51] activated p53 in human glioblastoma U87MG cells, as demonstrated by translocation of p53 from cytoplasm to nucleus accompanied with accumulation of total p53 expression. In line with our study, activation of p53 by COX inhibitors has also been demonstrated in colon and oral cancer cells [33,34,52]. We investigated whether celecoxib-induced p53 activation is followed by cell cycle arrest, apoptosis or autophagy in human glioblastoma cells. One study demonstrated a tumour cell-type dependent effect of cell cycle arrest and apoptosis following celecoxib treatment. Liu and colleagues (2003) reported that celecoxib-induced DNA damage led to G_2_M cell cycle arrest in mammary (and not lung) cancer, but apoptosis in lung (and not mammary) cancer cells [26]. The underlying mechanisms for these differential celecoxib-induced functional responses were not addressed. Our study in human glioblastoma cells reveal that celecoxib-induced p53 activation is followed by p53-dependent G_1 _cell cycle arrest and p21 activation. Celecoxib-induced G_1 _cell cycle arrest accompanied by increased p21 protein expression has been reported in human cholangiocarcinoma [12], colorectal [13,17], hepatocellular [35] and prostate cancer cells [14].

While apoptosis is considered a major anti-proliferative mechanism of celecoxib [9,13-15,17,26], our findings show that induction of p53-dependent G_1 _cell cycle arrest by celecoxib is followed by p53-dependent cell autophagy and not apoptosis. It should be noted that higher concentrations of celecoxib (40–100 μM) induce apoptosis [9,13-15,17,26]. The celecoxib concentrations (40–100 μM) are 4 to 11-fold greater than 8 μM, the human plasma concentration of celecoxib after consumption of 800 mg/kg per day [39] and the concentration that is presently used in this study. Mazzanti et al. (2009) recently showed that celecoxib (10 and 20 μM) induces apoptosis, but lower concentrations of celecoxib (at 2.5 and 5 μM) induce autophagy in hepatocellular carcinoma cells that are cultured in serum-free medium [35]. The sensitivity of tumour cells to celecoxib-induced cellular apoptosis or autophagy is likely to be concentration- or tumour type-dependent. The role of p53 in autophagy remains controversial with studies suggesting activation of p53, as well as inhibition of p53, as inductive of autophagy [46]. In our study, induction of autophagy by celecoxib in glioblastoma cells is p53-dependent, as shown by the autophagy induction only in celecoxib-treated glioblastoma cells with high (and not low) functional level of p53. In contrast, Mazzanti et al. (2009) reported that induction of autophagy by celecoxib (2.5 and 5 μM) is mediated by P-glycoprotein and Bcl2 via a p53-independent mechanism. The role of autophagy in cancer development is complex, as it has been implicated in both tumour survival and tumour cell death [53]. Induction of cell cycle arrest preceding autophagy induction inhibits tumor growth [54]. Our results support the induction of p53-dependent G_1 _cell cycle arrest, followed by autophagy as a mechanism for celecoxib to prevent glioma cell survival. Induction of p53-dependent autophagy independent of apoptosis should be considered as one of the underlying anti-proliferative mechanisms of COX-2 inhibitors, celecoxib in particular, in various tumours.

We investigated the 'up-stream' mechanisms preceding p53 activation in U87MG cells treated with celecoxib. We found that celecoxib (8 and 30 μM) induced DNA damage, accompanied with inhibition of DNA synthesis in U87MG cells, which led to p53-induced G_1 _cell cycle arrest and autophagy events. These findings of celecoxib-induced DNA damage followed by p53-dependent G_1 _cell cycle arrest and autophagy are clinically relevant since low concentration of celecoxib (8 μM) are attainable in human serum [39]. In cancer cells, DNA damage was induced following celecoxib treatment (at high concentrations of 50 and 100 μM) in murine lung (A549) and mammary (MCa-35) cancer cells [26], and by the non-selective COX inhibitor aspirin in HT-29 human colon carcinoma [25]. Activation of DNA damage-p53 signalling by COX-2 inhibitors has not been reported. One study proposes induction of DNA damage by the COX inhibitor R-flurbiprofen following the observation that R-flurbiprofen increases p53 (Ser 15) phosphorylation in colon cancer cells, but this has yet to be verified [33]. Our study demonstrates that selective COX-2 inhibition by celecoxib induces DNA damage and inhibits DNA synthesis, resulting in p53 activation and subsequent anti-proliferative effects in glioblastoma cells. The mechanisms underlying celecoxib-induced DNA damage remain unclear and are beyond the scope of this study. Whilst inhibition of COX-2 expression is reported to reduce generation of reactive oxygen species and prevent DNA damage, recent studies show that COX-2 inhibitors celecoxib [55] and sulindac [56], induce reactive oxygen species to mediate anti-tumour responses. Seo et al. (2007) also showed that induction of reactive oxygen species by sulindac was accompanied by phosphorylation of p53 (Ser 15) and accumulation of p53 in human multiple myeloma cells [56]. It is possible that celecoxib induces reactive oxygen species, followed by activation of DNA damage-p53 signalling to mediate anti-glioblastoma effects, but this requires further investigation.

## Conclusion

Our study reveals an important underlying mechanism of celecoxib-mediated inhibition of glioblastoma cell growth, by induction of DNA damage leading to p53-dependent G_1 _cell cycle arrest and autophagy, but not apoptosis. These results highlight the importance of p53 for enhanced anti-glioblastoma response by celecoxib. With the clinical relevant concentration of celecoxib used in this study, the present findings support potential clinical application of celecoxib to improve therapy of glioblastoma multiforme patients.

## Methods

### Cell culture and drug treatment

Human glioblastoma cells U87MG, U373MG, LN229 (American Type Culture Collection, Rockville, MD) and U87MG-E6 (U87MG cells transfected with human papillomavirus E6 oncoprotein, kindly provided by Russell O. Pieper, UCSF Comprehensive Cancer Centre, San Francisco, CA) were grown in Dulbecco's modified Eagle's medium (DMEM) supplemented with fetal bovine serum (FBS, 10%), nonessential amino acids (100 μM), sodium pyruvate (1 mM), streptomycin (100 μg/ml) and penicillin (100 U/ml, Gibco BRL, Grand Island, NY) at 37°C in an atmosphere containing 5% CO_2_.

Celecoxib (Pfizer Pharmacia, New York, NY) and pifithrin-α (PFT, Sigma, St. Louis, MO) was prepared as 100 mg/ml and 10 mg/ml stock in dimethyl sulfoxide (DMSO; Sigma), respectively. Stock solutions were diluted to required concentrations with culture medium on the day of treatment. U87MG cells were treated with PFT (U87MG-PFT) for 30 minutes prior to celecoxib treatment. Vehicle DMSO was used as drug replacement in experimental controls. The final DMSO concentration did not exceed 0.15% (v/v). All experiments were performed in accordance with guidelines approved by the Institutional Review Board of National Cancer Centre, Singapore.

### Cell viability assay

In 96-well plates, cells were treated with increasing concentrations of celecoxib to identify dose-dependent viability of U87MG, U87MG-E6, U87MG-PFT, LN229 and U373MG cells. In some cases, U87MG cells were pre-treated with PFT (25 μM) for 30 minutes prior to celecoxib treatment. After 24 and 72 hours, cells were stained with 3-(4,5-dimethylthiazol-2-yl)-2,5-diphenyltetrazolium bromide (MTT, 1 mg/ml), incubated for 4 hours at 37°C, lysed with lysis buffer (N, N-dimethyl formamide, 50%; sodium dodecyl sulphate, 20%) and absorbance measured at 570 nm. Readings of celecoxib-treated cells were normalised against DMSO-treated controls.

### Western blot analysis

Cells treated with DMSO or celecoxib (8 and 30 μM) were lysed and protein quantitated by Bradford assays (Bio-Rad Laboratories, Hercules, CA). Equal amounts of protein were separated in SDS-polyacrylamide gels and transferred onto nitrocellulose membranes (Amersham Biosciences). Membranes were blocked with 5% skim milk, incubated overnight (4°C) with monoclonal anti-p53 (DO-1, 1:500, Santa Cruz Biotechnology, Santa Cruz, CA) or rabbit polyclonal anti-LC3 (1:500, Santa Cruz Biotechnology), followed by horseradish peroxidase-conjugated secondary antibodies (Amersham Biosciences). Protein bands were visualised with ECL plus chemiluminescence kit (Amersham Biosciences). For loading controls, membranes were stripped and re-probed with horseradish peroxidase-conjugated anti-β-actin (Santa Cruz Biotechnology).

### Immunocytochemistry

Celecoxib-treated (0, 8 and 30 μM) cells (grown on cover slips) were fixed and permeabilised in 0.2% Triton X-100. After wash, cells were blocked with 5% BSA, incubated with specific antibodies against p53 (1:50, Neomarkers, Fremont, CA) or p21 (1:100, Santa Cruz Biotechnology) for 1 hour at room temperature, followed by incubation with anti-mouse FITC-conjugated secondary antibodies (1:200, Molecular Probes). Cover slips were mounted with VectaShield Mounting Medium containing DAPI (Vecta Laboratories, Burlingame, CA). Images were viewed under a Laser Scanning Microscope and images captured using software LSM510 (Zeiss, Germany).

### Cell cycle analysis and p21 mRNA expression

Cells were synchronised at G_o _phase in serum-free medium for 48 hours, followed by celecoxib treatment (0, 8 and 30 μM) in medium containing 10% FBS for 18 hours. In some cases, U87MG cells were pre-treated with PFT (25 μM) for 30 minutes prior to celecoxib treatment. For cell cycle analysis, collected cells were fixed overnight with ice-cold ethanol (75%), stained with propidium iodide (5 μg/ml, Sigma) supplemented with 100 μg/ml RNase (Sigma), and then analysed with flow cytometry using CellQuest Pro (BD Biosciences, San Jose, CA) for 10,000 events. For p21 mRNA analysis, total RNA was extracted from celecoxib-treated cells with Tri-Reagent (Molecular Research Centre, Cincinnati, OH). 1 μg total RNA was reverse transcribed using the ImProm-II Reverse Transcription system (Promega Corporation, Madison, WI). PCR was performed with specific primers for p21 (forward: 5'-CAGCATGACAGATTTCTACCAC-3', reverse: 5'-CCAGGGTATGTACATGAGGAG-3') and GAPDH (forward: 5'-GGAAGGTGAAGGTCGGAGTC-3', reverse: 5'-GTCTTCTGGGTGGCAGTGAT-3') at the following conditions: 94°C, 55°C and 72°C at 30 seconds each temperature for 30 cycles.

### Apoptosis and autophagy assays

Cells were treated with DMSO or celecoxib (8 and 30 μM) for 72 hours. In some cases, U87MG cells were pre-treated with PFT (25 μM) for 30 minutes prior to celecoxib treatment. For apoptosis assays, trypsinised cells were incubated with FITC-conjugated Annexin V (BD Biosciences) and propidium iodide (5 μg/ml). 10,000 events were analysed for apoptosis by FACS with CellQuest Pro software. For autophagy assays, celecoxib-treated cells were stained with acridine orange (1 μg/ml, Sigma) for 15 mins at 37°C. Trypsinised cells were re-suspended in phenol red-free growth media (Gibco BRL) and 10,000 events were analysed by FACS with CellQuest Pro software. Acridine orange-stained cells grown on cover slips were viewed under a Laser Scanning Microscope and images captured using software LSM510.

### Comet assays and ^3^H-thymidine incorporation assays

DNA damage was analysed by Comet assays as follows: Sub-confluent cells were treated with DMSO or celecoxib (8 and 30 μM) for 5 and 18 hours. Cells were mixed with 0.5% low melting point agarose and allowed to solidify on slides. Slides were immersed in lysis buffer, electrophoresis in tris-base buffer, stained with SYBR Green 1 (Trevigen, Gaithersburg, MD) and analysed with fluorescence microscopy. DNA damage, characterised by formation of comet tails, was quantitated by tail moments (multiplication of DNA tail migration from cell nucleus with DNA content) using Comet Score Freeware (Tritek Corporation, Summerduck, VA).

DNA synthesis was quantified with ^3^H-thymidine incorporation assays as follows: Sub-confluent cells were labeled with [methyl-^14^C]-thymidine (Amersham Biosciences, Buckinghamshire, UK) overnight, followed by celecoxib treatment (8 and 30 μM, 5 hours). After wash, cells were incubated with medium containing [methyl-^3^H]-thymidine (Amersham Biosciences) for 20 minutes, followed by 5% trichloroacetic acid and then 100% ethanol. Cells were air-dried, lysed in 1% sodium dodecyl sulphate and 10 mM NaOH, and subsequently the radioactivity measured with a liquid scintillation counter (Wallac, Turku, Finland). A control sample labelled with [methyl-^14^C]-thymidine alone was included to determine [methyl-^14^C]-thymidine signal spillover into [methyl-^3^H]-thymidine channel. DNA synthesis was presented as a percentage of [methyl-^3^H]-thymidine/[methyl-^14^C]-thymidine ratio in celecoxib-treated cells over controls.

### Statistical analysis

All values are presented as mean ± standard error of mean (S.E.M.) and *n *indicates number of independent experiments. The concentration-dependent effect of celecoxib on viability of glioblastoma cells was analysed by 2-way analysis of variance (2-way ANOVA). The effect of celecoxib on DNA damage, DNA synthesis, cell cycle progression, autophagy and cell apoptosis were analysed by 1-way ANOVA, followed by Dunnett's test (control vs. celecoxib, 8 or 30 μM). The effect of PFT on cell apoptosis and autophagy in U87MG cells was analysed by Mann-Whitney U-test. A *P *value of less than 0.05 was considered to be significant. GB-STAT (Dynamic Microsystems, Silver Springs, MD) statistical package was used in all calculations.

## Abbreviations

ANOVA: analysis of variance; AVO: acidic vesicular organelle; BSA: bovine serum albumin; COX-2: cyclooxygenase-2; DMSO: dimethyl sulfoxide; FBS: fetal bovine serum; FDA: Food and Drug Administration; LC3: light chain-3; PFT: pifithrin-α.

## Competing interests

The authors declare that they have no competing interests.

## Authors' contributions

KBK, SKY and QHG performed the experiments. KBK drafted the manuscript, analysed and interpreted the experimental findings. KBK and ZCJ conceived the study and participated in experimental concept and design. KBK, ZCJ and WMC wrote the final version of manuscript. All authors read and approved the final manuscript.
